# Synthesis and bioactivity of oxovanadium(IV)tetra(4-methoxyphenyl)porphyrinsalicylates

**DOI:** 10.1186/s13065-019-0523-9

**Published:** 2019-02-04

**Authors:** Gauri Devi Bajju, Ashu, Altaf Ahmed, Gita Devi

**Affiliations:** Department of Chemistry, University of Jammu, New Campus, Baba Sahib Ambedkar Road, Jammu, Jammu and Kashmir 180 006 India

## Abstract

Herein, we report the synthesis of metal complexes of vanadium with heterocyclic tetradentate ligand. Four N atoms of the heterocyclic porphyrin ring occupy the equatorial position and O atom of salicylic acid occupies the axial position in the complex. The thermal and chemical stability of the complexes were assessed by thermogravimetric analysis (TGA). The electrochemical behavior of the designed complexes is also studied using cyclic voltammetry. These complexes were then further evaluated for in vitro anticancer effects, anti-oxidant and behavior during acute toxicity of the synthesized porphyrin ligands and their oxovanadium(IV) complexes. The interaction of these metal complexes with radical scavenger 2,2-diphenyl-1-picrylhydrazyl (DPPH), encouraged us to study the anti-oxidant behavior of such complexes. The complex (SSA)VO(TMP) i.e. oxovanadium(IV)tetra(4-methoxyphenyl)porphyrinsulphosalicylate showed in vitro cytotoxic activity against glioblastoma (T986). It would be pertinent to mention here that the complex also did not exhibit any adverse toxicological symptoms and mortality in the target animal at the limit test dose level of 2000 mg/kg body weight.

## Introduction

The architectural alignment of complexes of macrocyclic ligands especially with a polydentate character has attracted tremendous attention by the researchers due to their ability to enclose plethora of metal ions and have found tremendous applications in varied areas of catalysis, medicine, solar-energy transformation and conductive substances [1, 2]. Novel porphyrin complexes are designed either by the coordination at the center of the porphyrin ring by different metal ions or by using peripheral substituted porphyrin rings with varied functional groups. This opens a route for various synthetic modifications and programming. Owing to the vast study dedicated in this field, many efforts have been devoted to the preparation of a variety of metalloporphyrin compounds that bear reformative characteristics with possible practical applications. However, to the best of our knowledge, very few reports summarize the biological activities of such metalloporphyrin complexes. Among various metalloporphyrins, vanadium porphyrins merit special attention because they are known as a new class of anti-human immunodeficiency virus (HIV) agents. Compared with other vanadium complexes as potential therapeutics, vanadium porphyrins are stable and rarely demetalated [3].

The above mentioned details about these complexes inspired us to investigate the synthesis and biological activities of vanadium metallic complexes. The synthesized complex oxovanadium(IV)porphyrin salicylate has no literature precedence with respect to its synthesis and biological studies. We began this study by first synthesizing it from metallated meso-tetra (*p*-methoxyphenyl) porphyrin ligand. The axially ligated oxovanadium(IV)porphyrin complexes were then evaluated for their biological activity including anti-cancer, anti-oxidant and effects against acute toxicity. In accordance with the structure confirmation, we also characterized the complex by nuclear magnetic esonance (NMR), infra red (IR), powder X-ray diffraction and mass spectroscopy.

## Materials and instruments

Standard procedures for the purification of reagent grade solvents have been already established and are available in free source and the same have been followed in our research also. Chemicals used in our research including vanadyl sulphate were purchased from Loba Chemie, and salicylic acid (SA) and sulfosalicylic acid (SSA) were purchased from Qualigens Chemicals and were used as received. These chemicals were that of reagent grade. Pyrrole (Fluka, Switzerland) was distilled at room temperature over potassium hydroxide (KOH) pellets under reduced pressure before use. *p*-Anisaldehyde (*p*-methoxylbenzaldehyde) (Aldrich, USA) and propionic acid (Qualigens, India) were used as supplied. *N*-tetrabutylammonium hexafluorophosphate (TBA)PF6 was recrystallized twice from ethyl acetate and dried prior to use Estimation of vanadium was done using the gravimetric analysis (based on its mass) and it came out as silver orthovanadate. VO(TMP) i.e. meso-tetra(*p*-methoxyphenyl)porphyrin)oxo-vanadium(IV) used for the synthesis of axially ligated complexes was synthesized according to the procedure as given in literature [4].

We used CHNS analyser CHNS-932 for performing the micro analysis of carbon, hydrogen, nitrogen and sulphur. We used Perkin Elmer grating spectrophotometer using KBr discs for observing and recording the corresponding IR spectra of the complexes, spread over the region of 4000–400 cm^−1^. Electronic spectra of the complexes were run in CDCl_3_ on a Perkin Elmer Ultraviolet visible spectrophotometer in the range of 200–600 nm by using 10^−4^ M solution of the complexes. For recording the ESI Mass spectra, we used a Bruker Daltonics mass spectrophotometer using the positive linear high power of detection settings at an accelerating voltage of 20 kV and laser power tuned depending on the sample. We used a Bruker Avans 400 MHz spectrophotometer for recording the ^1^H NMR spectra. TGA and DTA (differential thermal analysis) were recorded on Linseis STA PT-100 thermometer using dry samples at the heating rate of 10 °C/min in an air atmosphere. The TG–DTA results were recorded in argon atmosphere from room temperature to 900 °C. XRD was recorded on X’Pert Pro XRD employing CuKα. radiation (λ = 1.541 Å) in the range 10°–70° at Panjab University, India.

The CV (cyclic voltammetric) measurements were carried out by an Autolab Computer-controlled electrochemical measurement system equipped with a potentiostat. A three electrode system comprised a gold working electrode, a platinum wire counter electrode, and a saturated Ag/AgCl in KCl as reference electrode. A 0.1 M solution of (TBA)PF_6_ in freshly distilled CH_2_Cl_2_ was used as a supporting electrolyte during the electrochemical experiments. The scan rate was 20 mV/s and the range was − 0.2 to 0.2 mV. Concentration of the complexes was 10^−6^ M. The solution were purged with oxygen free nitrogen gas prior to measurements and all measurements were made at room temperature.

## Biological studies

### Antioxidant studies

#### 1,1-Diphenyl-2-picrylhydrazyl (DPPH) radical scavenging assay

Radical scavenging activity (RSA) was assessed using a purple colored methanol solution of DPPH radical which was first bleached and then measured for free radical rummage. The radical rummage was determined according to the reported method of Blois et al. with modifications. A mixture consisting of 1 ml 0.5 mM methanol solution of the DPPH radical, 2 ml of the complex sample and an equal quantity (2 ml) of 0.1 M sodium acetate buffer at pH of 5.5 was stirred at room temperature in dark surroundings for half an hour. The absorbance of the mixture was measured using UV–Vis spectrophotometer at 517 nm as standard wavelength. The free radical rummage was calculated as a percentage of DPPH radical discoloration, using the standard established equation.

$$\% {\text{RSA}}\, = \,\left[ {\left( {{\text{A}}_{0} \, - \,{\text{A}}_{\text{s}} } \right)/{\text{A}}_{0} } \right]\,\; \times \,\;100.$$

where A_0_ is the absorbance of the control and A_s_ is the absorbance of the test compound. The effective concentration of sample required to scavenge the corresponding radical by 50% (IC50 value) was obtained by linear regression analysis of the curve plotting between % RSA and concentrations.

### In vitro cytotoxicity against human cancer cell lines

#### Cell lines and cell cultures

The lung (A-549), glioblastoma (T98G) and human prostate (PC-3) cell line were grown and maintained in RPMI-1640 medium (Roswell Park Memorial Institute medium), pH 7.4, whereas Dulbecco’s Modified Eagle Medium (DMEM) was used for breast (T47D). The media were supplemented with paclitaxel (1 μM), mitomycin (1 μm), adriamycin (1 mm) and the cells were allowed to grow in CO_2_ incubator (Heraeus, GmbH, Germany) at 37 °C with 90% humidity and 5% CO_2_ atmosphere. The cells were then exposed to the solution of metal complexes in DMSO as solvent, while the untreated control cultures received only the vehicle (DMSO, < 0.2%).

### Cytotoxicity assay

The standard sulforhodamine B dye assay was used to determine the In vitro cytotoxicity against human cancer cell lines wherein both of the test samples were prepared in stock solutions in dimethyl sulfoxide (DMSO) [5, 6].

### Experimental animals

Female Wistar albino rats (ranging from 10 to 12 weeks ages) were used for our analysis. The animals were housed five per cage in the polypropylene cages in the animal house. All of them were kept in an experimental setup with proper ventilation (more than 10 air changes per hour) which facilitated clean and fresh air. A light/dark photo transition period with a duration of 12 h was maintained. Room temperature and relative humidity were also maintained between 22 ± 2 °C and 40–80% respectively according to Committee for the purpose of control and supervision of experiments on animals (CPCSEA) guidelines. The animals had free access to pelleted feed. Standard water for our analysis was used from (uv + uf model). The animals were examined at intermittently for behavioural abnormalities if any. No animals were harmed during this analysis strictly in accordance with the protocol approved by the Institutional Animal Ethics Committee.

### Acute toxicity study

The acute toxicity study of (SA)VO(TMP) and (5-SSA)VO(TMP) was carried out in female Wistar rats according to fixed dose procedure (The Organisation for Economic Co-operation and Development, OECD Guideline No. 420). Five female rats received dose of 2000 mg/kg body weight, orally after a 16 h fasting period using an oral gavages.

Observations were recorded after administering the dosages for gross general behavior of the animals (like gait, skin color, lacrimation, sleepiness, writhing etc.) interminable for the first hour, with focused recording during the first 4 h, followed by the periodical recording in the next 20 h (total one full day) and thereafter the same behavioral patterns recorded for a period of 2 weeks for mortality and any other observable toxicity. Finally, the number of survivors with their behavioral patterns were recorded.

### Cage-side observations

Hand–eye coordination, any changes in the eyes and mucous membranes, fur, skin, respiratory and circulatory changes, autonomic and any changes in the nervous systems were recorded in detail after the studies and also movements of their bodies and their behavioural patterns. Observations of convulsions, tremors, diarrhea, salivation, lethargy, sleep, and coma were specifically recorded. All rats were observed for toxic signs and any pre-terminal deaths daily.

### Necropsy

After the experimental studies were over, we studied all the animals for their detailed gross necropsy which includes minute and careful observation of the bodies’ superficial surfaces, all apertures and the cranial, thoracic and abdominal cavities.

### Data calculation using statistical analysis

Statistical analysis in all the above toxicity studies was performed using Primer software. For best correlation all results were expressed as standard mean ± S.E.M. i.e. standard error of mean. Data were analyzed using one-way analysis of variance (ANOVA), and when appropriate Student–Newman–Keul’s multiple comparison test of treated group with vehicle control mean was done.

## Synthesis of axially ligated VO(IV) porphyrins, (5-XSA)VO(TMP) where X = H and SO_3_H

**Fig. 1:**
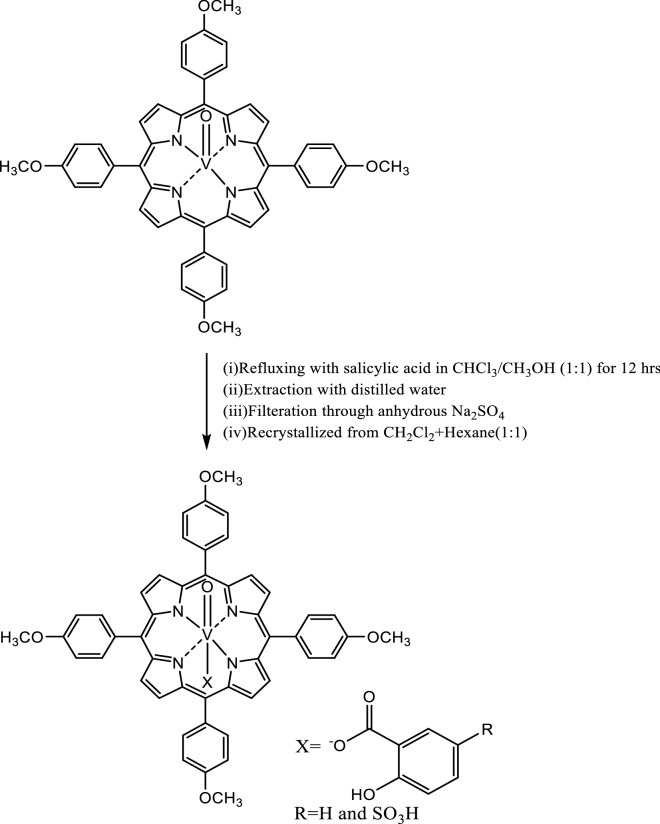
Synthesis of axially ligated complexes

VO(TMP) (0.15 mmol) in 25 ml CHCl_3_ was treated with appropriate salicylate (0.56 mmol) in 25 ml CH_3_OH and refluxed. After concentration, the mixture was dissolved in minimum quantity of dichloromethane (DCM) and extracted with distilled water to remove excess salicylic acids. The DCM layer was collected and dried over anhydrous Na_2_SO_4_. The compound was recrystallized using DCM-hexane solution in the ratio of 1:1. The same procedure was applied for the synthesis of sulphosalicylic acid axially ligated oxovanadiumporphyrin complex. The synthetic scheme representing the proposed structure is shown as Fig. 1. Yield of the purified axially ligated oxovanadiumporphyrin complexes in the range of 45% was recorded. Figure 1 represents the synthesis of axially ligated complexes.

### Spectroscopic characterization of the complexes

- Oxovanadium(IV)porphyrinsalicylate complexes: UV–Vis(CHCl_3_): λ_max_ (in nm) (log ε) 447.9 (4.712) for *B*-band and 560.7 (4.248) and 652.1 (4.106) for Q-bands; IR (KBr, cm-): 967.34 (V=O); ^1^H NMR (CDCl_3_): *δ* 8.89 (m, 8H, *β*-pyrrole protons), 8.14 (m, 8H, H_o_), 7.30 (m, 8H, H_m_.), 7.74 (m, 4H) for salicylate aryl protons, 4.12 (m, 12H, Ho_CH3_); m/e: 936.24; elemental analysis: C_55_H_41_N_4_O_8_V: calculated data: C 70.51; H 4.41; N 5.98; S 0.00; observed data: C 69.99; H 4.12; N 5.11; S 0.00. vanadium estimated: (Calc.)V 5.44; (Obs.)V 5.01.
- Oxovanadium(IV)porphyrinsulphosalicylate complexes: UV–Vis(CHCl_3_): λ_max_ (in nm) (log ε) 452.0 (4.0 12) for *B*-band and 565.1 (4.048) and 659.1 (4.006) for Q-bands; IR (KBr, cm-): 977.91 (V=O); ^1^H NMR (CDCl_3_): *δ* 8.76 (m, 8H, *β*-Pyrrole Protons), 8.45 (m, 8H, H_o_), 8.14 (m, 8H, H_m_.), 7.65 (m, 3H) for sulphosalicylate aryl protons; m/e: 1016.19; elemental analysis: C_55_H_41_N_4_O_11_V; calculated data: C 64.96; H 4.06; N 5.51; S 3.15; observed data: C 63.06; H 3.16; N 5.11; S 3.01; vanadium estimated: (Calc.)V 5.01; (Obs.)V 4.98.

## Results and discussion

The characteristic B and Q bands in the range of 410–420 and 450–690 nm, respectively are reflected in the electronic absorption spectra as the typical patterns of porphyrin metal complexes [7]. After the insertion of the metal ion into the porphyrin ring, there was a general tendency of decrease in the intensity as well as the number of Q bands and a slight blue shift was observed in the B (soret) band. In axially ligated oxovanadium(IV)porphyrin complexes both B and Q band regions of the spectra show slight red shift indicating salicylates coordination to form 6-coordinate complexes. The observed red shift and broadening of the bands are indicative of the highly distorted porphyrins in solution [8].

**Fig. 2:**
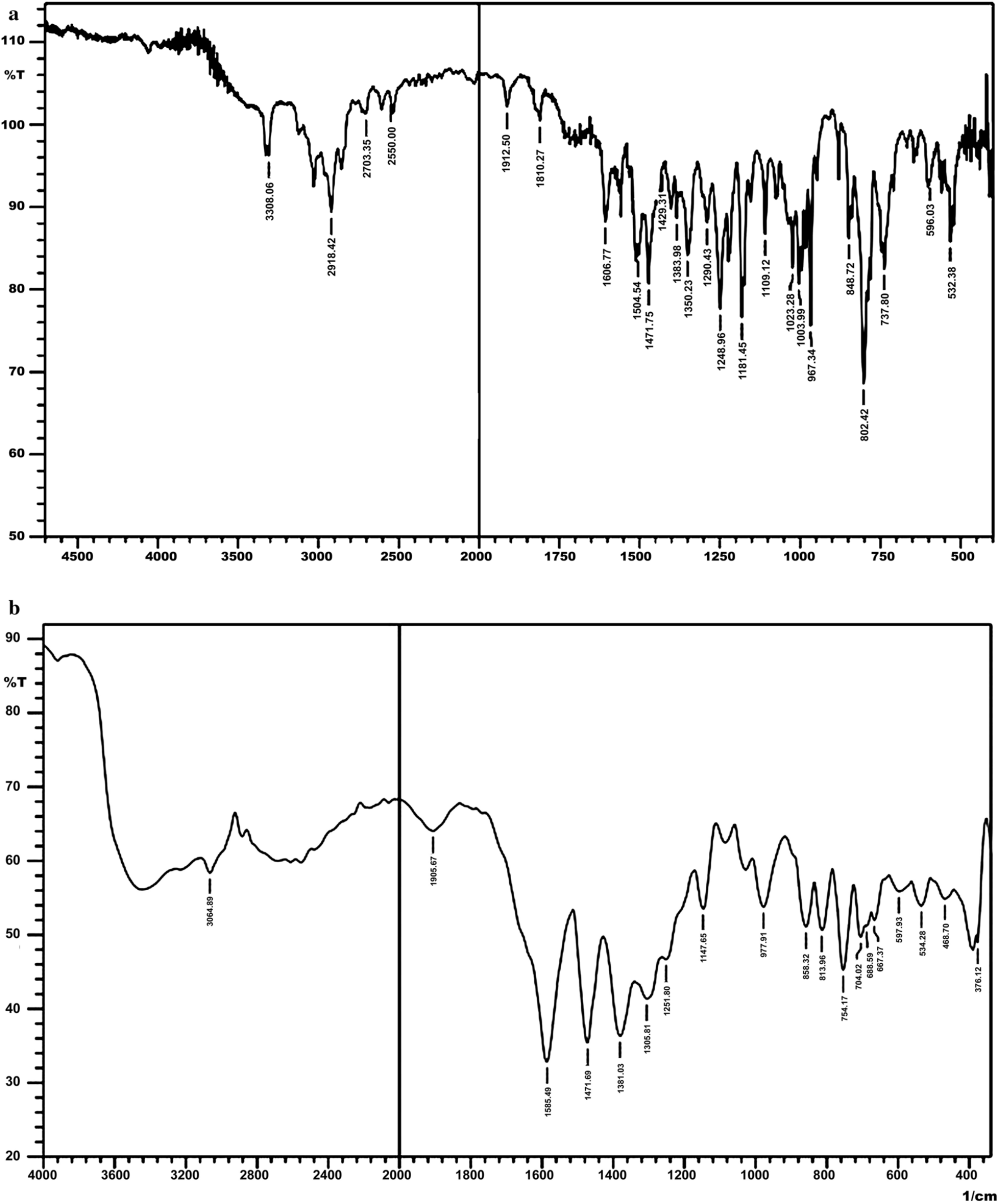
IR spectra of **a** VO(TMP)SA and **b** VO(TMP)SSA

On comparing the infrared spectral data of H_2_TMP and its corresponding metal porphyrin precursor, it was found that the band at 3447 cm^−1^ in H_2_TMP assigned to ν(N–H) (pyrrole) stretching vibration was disappeared in metallated complexes [9] and the characteristic ν(V–N) vibration frequency was found at ~ 500–430 cm^−1^, which corroborated with the indication of inception of oxovanadium(IV)porphyrin compounds [10]. In the spectra of all the axially ligated oxovanadium(IV)porphyrin complexes the incorporation of salicylates in VO(IV) metal derivatives of different porphyrins i.e., (SSA)VO(TMP), is confirmed by the absence of ν(OH) of carboxylate group stretching frequencies in complexes confirms coordination of the salicylate ligand to the vanadium(IV) metal centre via the carboxylate anion. A broad band centered at 3327 cm^−1^ for complexes is attributed to the O–H stretch of the hydroxyl group of SA ligand. Moreover, the values of V=O are stretching frequencies are consistent with that reported for distorted octahedral oxovanadium(IV) species [11] (Fig. 2)

Thus, the oxovanadium atom in the centre of porphyrin ring coordinate with the salicylate group axially to form six-coordinate complex of VO(IV) porphyrin.

**Fig. 3:**
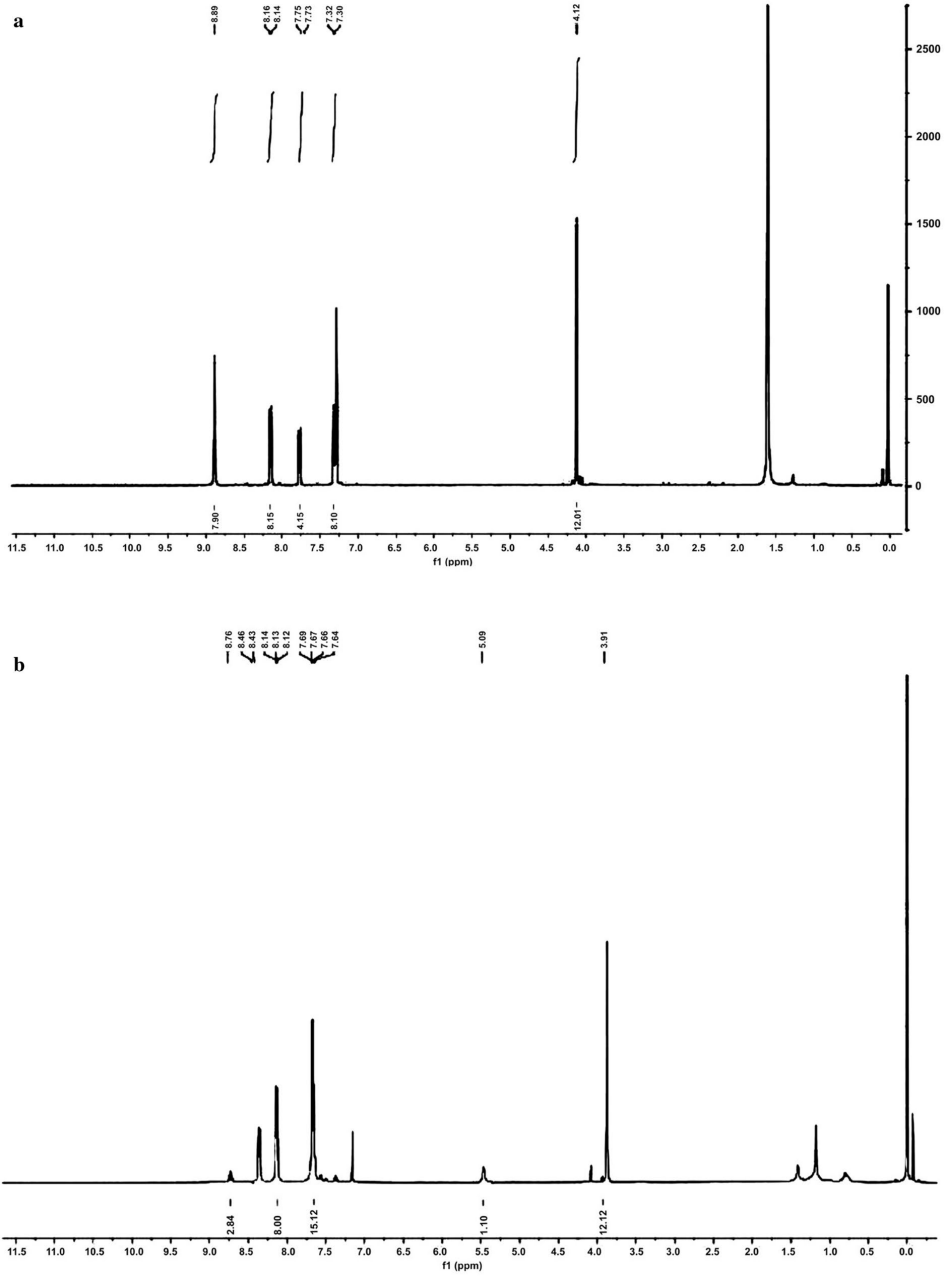
NMR spectra of **a** VO(TMP)SA and **b** VO(TMP)SSA

In all the metallated porphyrins there was absence of signal related to N–H protons of free H_2_TMP and shift in other signals indicating the insertion of oxovanadium in porphyrin macrocycle. The general tendency that reflects here is that the signals of axial salicylate protons shift to a higher field when compared to the signals of porphyrin protons and proton signals of free salicylic acid derivatives [12] in axially ligated oxovanadium(IV)porphyrin complexes (Fig. 3). This spatial distribution of protons prove that axial ligand is under the influence of π-conjugated system of porphyrin macrocycle.

Mass spectrometric characterization of (SSA)VO(TMP) complexes employed ESI as soft ionization technique. When we employed this technique, characteristic presence of molecular ion peaks was observed and recorded in the mass spectra of axially ligated oxovanadium(IV)porphyrins.

**Fig. 4:**
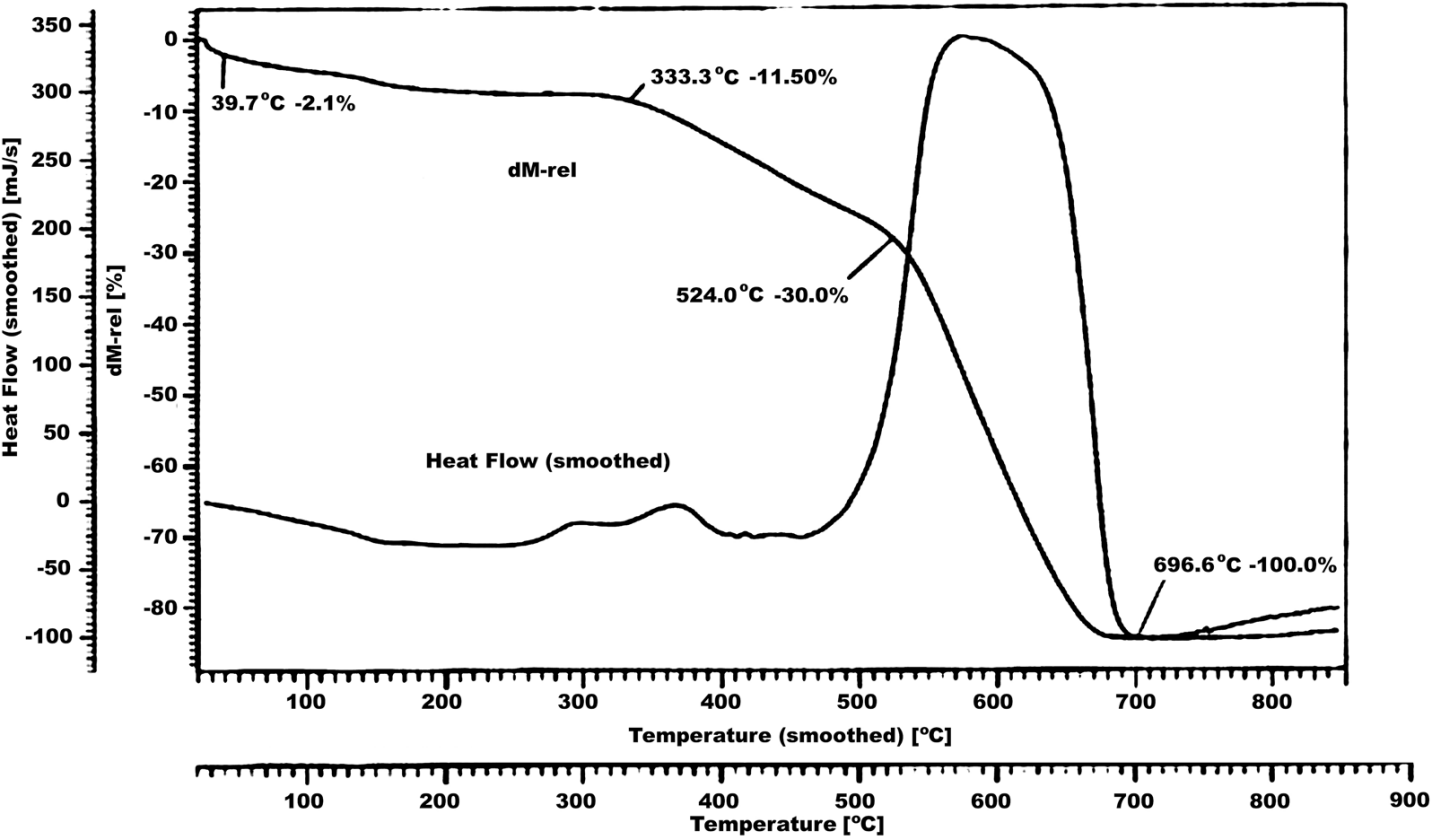
TG analysis of [VO(TMP)SSA])

The thermal stability in air of the complexes has been investigated by TG. From literature [13] it has been found that metallated complex is more stable than the corresponding porphyrin ligand. From the TG curve (shown as a descending curve in Fig. 2) of thermochemical analysis, it is clear that the complex is stable up to 330 °C approximately which illustrates the sample has an excellent stability. The weight loss of 2.1% observed between 20 and 40 °C is elimination of small molecular impurity. Then the weight loss (11.50%) observed at 333.3 °C is attributed to the removal of *p*-methoxyphenyl group (the theoretical value of 16.9%) and the weight loss (30.0%) at 524.0 °C shows the loss of rest of the three *p*-methoxyphenyl groups (the theoretical value of 34.5%). After 30% loss at 524 °C until 696 °C, the mass loss reaches 100% which is due to the complete disintegration of the complex. Simultaneously, there are some overlapped exothermal peaks on DTA curve (shown in Fig. 4 as ascending curve) in the range of 450–600 °C corresponds to the decomposition of porphyrin skeleton.

**Fig. 5:**
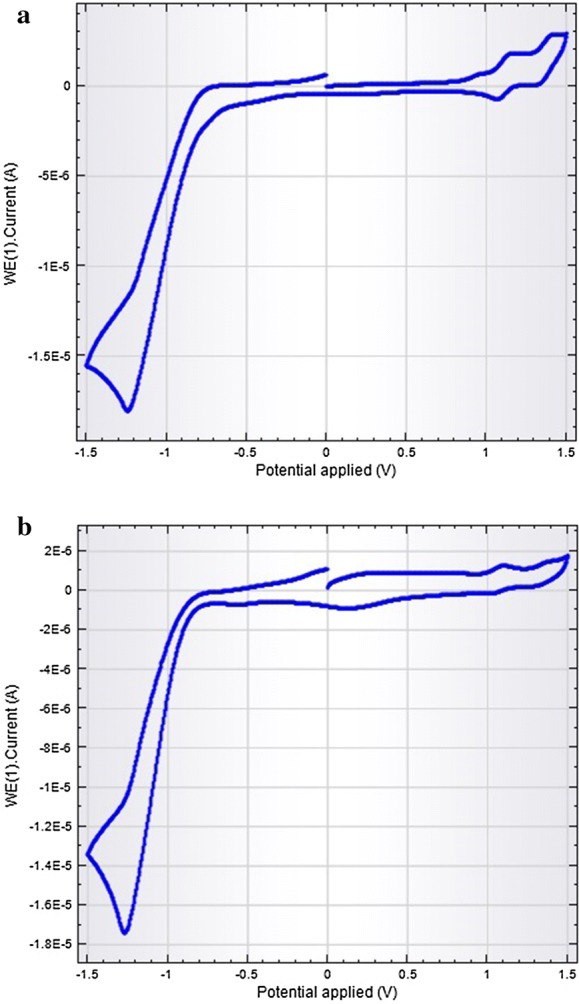
**a**, **b** Cyclic voltammetry studies

Electrochemical data obtained from cyclic voltammetry studies (shown in Fig. 5a, b) reveal that the synthesized complexes undergo two one-electron oxidations due to π-cation radical and dication formation and three one-electron reductions. The first two reductions are because of π-anion radical and dianion formation, while the third quasi-reversible reduction is assigned to a metal-centered process (V^IV^ → V^III^) tabulated in Table 1 All of the complexes reported are redox active in solution. Figure 5a shows cyclic voltammetry graph of [VO(TMP)SA] and 5b shows cyclic voltammetry graph of [VO(TMP)SSA]. Please note that [VO(TMP)SA] is oxovanadium(IV)tetra (4-methoxyphenyl)porphyrinsalicylate and [VO(TMP)SSA] is oxovanadium(IV)tetra(4-methoxyphenyl)porphyrinsulphosalicylate)

**Table 1:** Cyclic voltammetry studies of [VO(TMP)SA] and [VO(TMP)SSA] complexes

Species	E_OX2_/V	E_OX1_/V	E_red1_/V	∆E d/V
[VO(TMP)SA]	1.22	0.66	− 1.28	0.62
[VO(TMP)SSA]	1.25	0.68	− 1.30	0.62

**Fig. 6:**
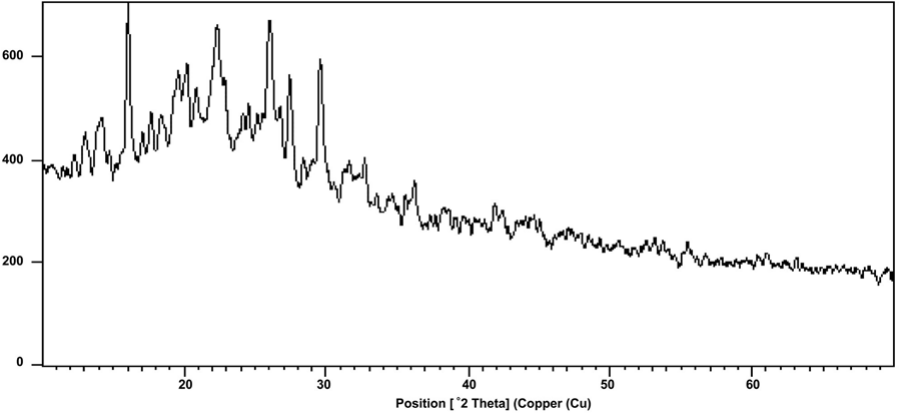
Powder X-ray spectrum of [VO(TMP)SSA]

Characterization of the complex [VO(TMP)SSA] was done at room temperature using the standard and shown in Fig. 6. X-ray diffraction technique by using Cu Kα radiation. The diffraction pattern of complex is recorded between 2θ ranging from 10° to 70°. The 2*θ* value with maximum intensity of the peak for the compound was found to be 16.092 (2*θ*) which corresponds to *d* = 5.52920 Å. The 2*θ* values for the prominent peaks have been found to be at 19.4205, 20.2249, 22.3301, 25.9619, 27.4460 and 29.6615 owing to the simple cubic phase (Fig. 6). We used the standard Scherrer’s formula for calculating the particle size of the sample. According to Scherrer’s equation, the particle size is given by t = 0.9 λ/Bcosθ, where t is the crystal thickness (in nm), B is half width (in radians), θ is the Bragg angle and λ is the wavelength. The particle size corresponding to each diffraction maxima are calculated using the measurements of the half width of the diffraction peak. We confirmed the particle size was well within the range of 23.15–50.11 nm for the complexes [14] in our studies.

### Antioxidant study

Primarily, DPPH is a stable free radical that is often used for the detection of radical rummage activity in chemical analysis. Antioxidants can induce a marked decrease in the absorption capability of DPPH radicals and the same was observed at 517 nm which shows the trends in their reduction capabilities. We got encouraging results from the antioxidant studies of axially ligated oxovanadium(IV)porphyrin complexes viz VO(TMP)(SA) and VO(TMP)(SSA) with IC_50_ values of 25 μg/ml and 40 μg/ml respectively as tabulated below (Table 2). These complexes showed remarkable rummage with radical rummage activity with lowest IC_50_ values whereas on the other hand our oxovanadium(IV)porphyrin VO(TMP) and porphyrin ligand TMP did not show such antioxidant behaviour.

**Table 2:** Antioxidant studies of porphyrin and its complexes

S. no.	Complexes	DPPH radical rummaging activity (IC50 µg/mL)
1	TMP	80
2	VO(TMP)	75.5
3	VO(TMP)(SA)	25
4	VO(TMP)(SSA)	40
5	SA	62
6	SSA	65

*TMP* tetra(4-methoxyphenyl)porphyrin, *VO(TMP)* oxovanadium(IV)tetra(4-methoxyphenyl)porphyrin *[VO(TMP)SA]* oxovanadium(IV)tetra(4-methoxyphenyl)porphyrin salicylate, *[VO(TMP)SSA]* oxovanadium(IV)tetra(4-methoxyphenyl)porphyrinsulphosalicylate

### In vitro cytotoxicity

The cytotoxicity of ligand (TMP), precursor [VO(TMP)], (SA)VO(TMP) and (SSA)VO(TMP) was evaluated against four human cancer cell lines of different tissues viz., lung (A-549), glioblastoma (T98G), prostate (PC-3) and breast (TA7D) at 1 × 10^−4^ M as shown in Table 3. Growth inhibition was calculated as a measure of percentage and observed against all the cancer cell lines. Among all the four compounds only the [VO(TMP)(SSA)] complex showed prominent in vitro cytotoxic activity against glioblastoma (T98G) cancer cell line, the percent growth inhibition was found to be 75% against glioblastoma (T98G) and 55% against prostate (PC-3) human cancer cell lines, respectively. Rest of the compounds TMP, VO(TMP) and (SA)VO(TMP) showed moderate percent growth inhibition i.e., in the range 13–45% against various cancer cell lines.

**Table 3:** Cytotoxicity activity of porphyrin and its complexes

Groups	(% growth inhibition)				
	Conc.	Lung (A549)	Glioblastoma (T98G)	Prostate (PC3)	Breast (T47D)
TMP	1 × 10^−4^ M	13	15	17	14
VO(TMP)	1 × 10^−4^ M	15	18	20	17
(SA)VO(TMP)	1 × 10^−4^ M	25	35	25	27
(SSA)VO(TMP)	1 × 10^−4^ M	45	75	55	27
SA	1 × 10^−4^ M	18	21	20	15
SSA	1 × 10^−4^ M	21	22	24	20
Paclitaxel	1 µM	65	–	–	–
Mitomycin-c	1 µM	–	–	68	–
Adriamycin	1 µM	–	59	–	73

*TMP* tetra(4-methoxyphenyl)porphyrin, *VO(TMP)* oxovanadium(IV)tetra(4-methoxyphenyl)porphyrin, *[VO(TMP)SA]* oxovanadium(IV)tetra(4-methoxyphenyl)porphyrinsalicylate, *[VO(TMP)SSA]* oxovanadium(IV)tetra(4-methoxyphenyl)porphyrinsulphosalicylate

### Acute toxicity of porphyrin complexes (SA)VO(TMP) and (SSA)VO(TMP)

#### Body weight, food and water intake

**Fig. 7:**
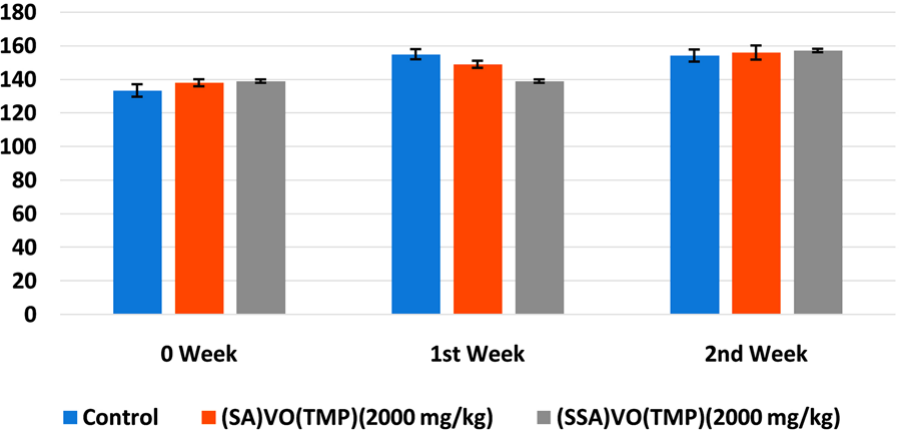
Mean body weight change of female Wistar rats

Individual body weights were recorded once a week as shown in Fig. 7. Figure 7 shows acute toxicity of [VO(TMP)SA] and [VO(TMP)SSA] showing mean body weight change of female Wistar rats. Food and water intake was recorded daily and average weekly consumption was calculated and data is shown in Table 4.

**Table 4:** Mean weekly feed intake and water intake of female Wistar rat treated

Groups	Feed intake		Water intake	
	1st week	2nd week	1st week	2nd week
Control	140 ± 3.1	152 ± 4.7	172 ± 4.3	189 ± 3.9
[VO(TMP)SA] (2000 mg/kg)	159 ± 2.2	161.5 ± 4.9	167 ± 2.1	185 ± 3.9
[VO(TMP)SSA] (2000 mg/kg)	158 ± 4.2	163 ± 5.1	169 ± 2.9	183 ± 4.6

Values are mean ± SEM (n = 5/group). Student’s t-test

*[VO(TMP)SA]* oxovanadium(IV)tetra(4-methoxyphenyl)porphyrinsalicylate, *[VO(TMP)SSA]* oxovanadium(IV)tetra(4-methoxyphenyl)porphyrinsulphosalicylate

### Hematology

**Fig. 8:**
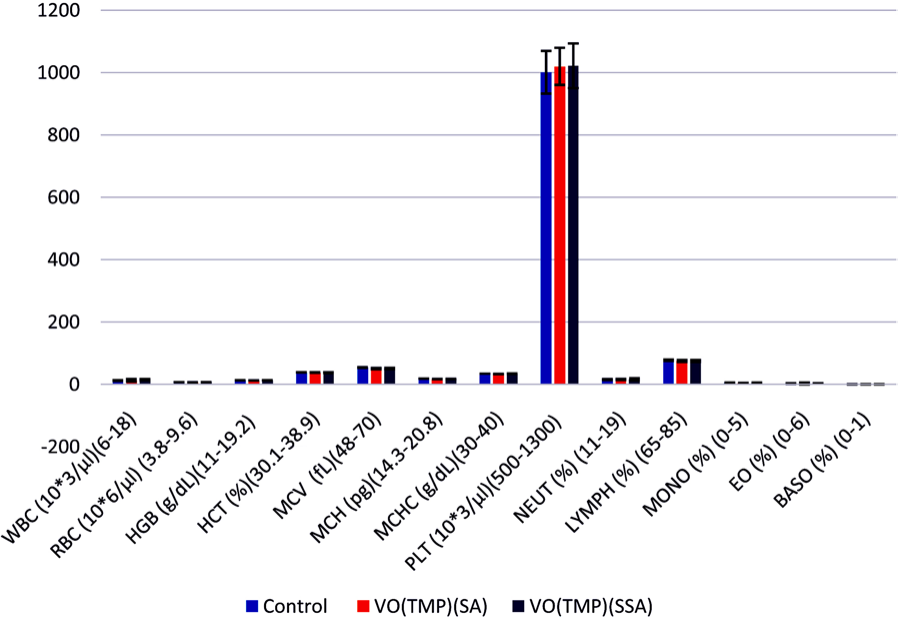
Mean hematological values of female Wistar rats

Blood was collected by retro orbital plexus from the overnight fasted animals. We took the services of an automated hematology analyser (make and model—Sysmex, XT-1800i, Kobe, Japan). Investigation of whole blood for the following was done: red blood cells (RBC), white blood cells (WBC), hemoglobin (Hb), platelet count, hematocrit (HCT), mean corpuscular volume (MCV), mean corpuscular hemoglobin concentration (MCHC), neutrophil (Neut), lymphocytes (Lymp), monocytes (Mono), eoisinophils (Eos) and basophils (Baso). A careful examination of animals show no abnormalities in the hematological parameters as shown in Fig. 8. Figure 8 shows acute toxicity of [VO(TMP)SA] and [VO(TMP)SSA] showing mean hematological values of female Wistar rats.

### Clinical biochemistry

**Fig. 9:**
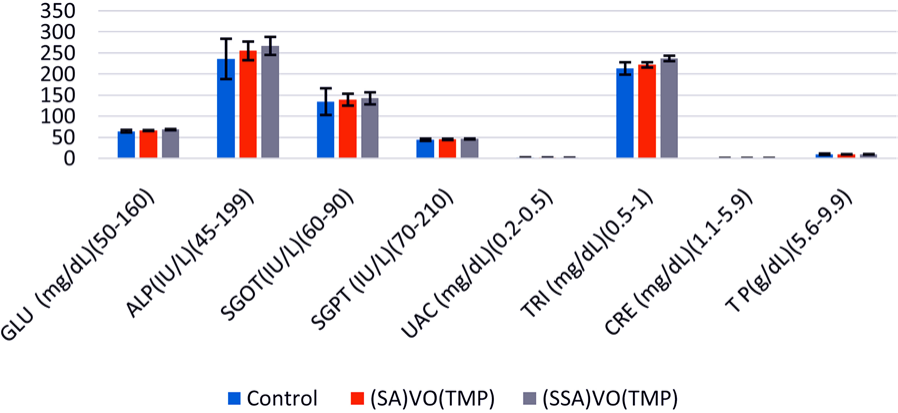
Mean biochemical values of female Wistar rats treated with complexes

After 14 days animals were fasted, blood collected in separate tubes without anticoagulant and allowed to coagulate, centrifuged at 2000 rpm for 10 min to obtain the serum. The serum was assayed for the determination of glucose, total protein, total bilirubin, alkaline phosphatase (ALP), ALT, AST, triglyceride, creatinine, urea, cholesterol by commercial kits procured from Transasia Bio-medicals Ltd India (Erba Manheim) using clinical biochemistry analyzer Chem-7 (Erba, Manheim, Germany) in the process as seen in Fig. 9. Figure 9 shows mean biochemical values of female Wistar rats treated with complexes [VO(TMP)SA] and [VO(TMP)SSA].

Thus from the above experimental study it is concluded that;

1. Both the complexes [VO(TMP)SA] and [VO(TMP)SSA] have not shown any toxicological symptoms and mortality at the limit test dose level of 2000 mg/kg body weight.
2. LD_50_ in female Wistar rats is more than 2000 mg/kg body weight.
3. Classified as category 5 as per GHS of OECD.

## Conclusion

The axially ligated oxovanadium(IV)porphyrin complexes with salicylates and its derivatives were synthesized and their in vitro antioxidant and anticancer potential was evaluated. Anticancer results obtained showed that the synthesized compounds have higher activity than the corresponding porphyrin ligand, but lower activity then the standard drug. The above synthesized compounds were characterized by well-established elemental analysis, ^1^H NMR, Mass spectroscopy, UV/visible, IR spectroscopy and Powder X ray diffraction technique. The octahedral geometry and simple cubic phase is proposed on the basis of these studies. In addition, (5-SSA)VO(TMP) has not shown any toxicological symptoms and mortality at the limit test dose level of 2000 mg/kg body weight.
